# Nonalcoholic fatty liver disease and mortality from all causes, cardiovascular disease, and cancer: a meta-analysis

**DOI:** 10.1038/s41598-019-47687-3

**Published:** 2019-07-31

**Authors:** Yan Liu, Guo-Chao Zhong, Hao-Yang Tan, Fa-Bao Hao, Jie-Jun Hu

**Affiliations:** 1 Department of Gastroenterology, The Fifth People’s Hospital of Chengdu, Chengdu, 611130 China; 2 Department of Hepatobiliary Surgery, The Second Affiliated Hospital of Chongqing Medical University, Chongqing, 400010 China; 3 Pediatric Surgery Center, Qingdao Women and Children’s Hospital, Qingdao University, Qingdao, Shandong 266034 China

**Keywords:** Health care, Non-alcoholic fatty liver disease

## Abstract

Whether nonalcoholic fatty liver disease (NAFLD) is associated with an increased risk of mortality remains controversial. The present study aimed to clarify this issue. A systematic search of PubMed and Embase was conducted through October 2018. Studies providing risk estimates of NAFLD and mortality were included. A random-effects model was employed to calculate summary risk estimates. Subgroup analyses were performed to identify potential effect modifiers. Fourteen studies, involving 498501 subjects and 24234 deaths, were included. Patients with NAFLD were found to be at an elevated risk of all-cause mortality compared with those without [hazard ratio (HR) = 1.34; 95% confidence interval (CI) 1.17–1.54)]. The significantly positive association between NAFLD and all-cause mortality could not be modified by age, sex, follow-up duration, and adjustment for body mass index, diabetes, smoking or hypertension (all *P*_interaction_ > 0.05), and remained in sensitivity analyses. No significant associations of NAFLD with CVD (HR = 1.13; 95% CI 0.92–1.38) and cancer (HR = 1.05; 95% CI 0.89–1.25) mortality were found. In conclusion, NAFLD is a predictor of increased all-cause mortality but not CVD and cancer mortality. These findings have important implications for decision making in public health and clinical practice, and highlight the urgency of developing effective treatments for NAFLD.

## Introduction

Nonalcoholic fatty liver disease (NAFLD) is regarded as the hepatic manifestation of metabolic syndrome, ranging from simple hepatic steatosis to nonalcoholic steatohepatitis (NASH). NAFLD has become a major cause of chronic liver disease worldwide, and is treated as a public health priority. A recent meta-analysis showed that the global prevalence of NAFLD had reached as high as 25.2%^1^, and this number is expected to be 33.5% in 2030^2^. Therefore, determining long-term outcomes, including morbidity and mortality, among NAFLD patients has important implications for decision making in public health and clinical practice.

In addition to its potential to induce cirrhosis and liver cancer, NAFLD is found to be a well-established risk factor for chronic kidney disease^3^, type 2 diabetes^4^, and cardiovascular disease (CVD)^5^. Hence, it is anticipated that NAFLD is a predictor of increased mortality. However, the results from observational studies on NAFLD and mortality remain controversial. Several studies found that patients with NAFLD were at an elevated risk of all-cause mortality compared with those without^6–9^, whereas other studies found no association between NAFLD and mortality^10–13^. Moreover, a recent large prospective study found that NAFLD was associated with an increased risk of death from all causes, CVD and cancer in women but not in men^14^. Two recent meta-analyses consistently showed that NAFLD was not associated with all-cause mortality^1,15^. However, these meta-analyses included only around one-third of studies currently available. More importantly, these meta-analyses pooled data from multiple reports that originated from the same cohort (i.e., the Third National Health and Nutrition Examination Survey cohort, NHANES III)^13,16–18^.

To our knowledge, a comprehensive meta-analysis focusing on NAFLD and mortality was not available. Therefore, we performed this study to investigate the associations between NAFLD and mortality from all causes, CVD and cancer.

## Methods

We reported our results in adherence to the Meta-analysis Of Observational Studies in Epidemiology (MOOSE) statement.

### Search strategy

To identify potentially eligible studies, we performed a systematic search of PubMed and Embase through October 2018, with no restriction. The search strategy applied in these two databases was provided in detail in Table S1. We also manually searched the reference lists of pertinent articles for identifying additional studies. We did not contact the original authors to obtain extra data parameters.

### Study selection

All follow-up studies that reported risk estimates and corresponding confidence intervals (CIs) on the association of NAFLD with mortality from all causes, CVD or cancer were eligible for inclusion. We only considered studies that used either imaging techniques or liver biopsy for diagnosing NAFLD. We did not impose any restrictions on the presence of comorbidity in the study population. Following studies were excluded: (1) studies using NAFLD patients as the reference group; (2) studies conducted in cancer patients undergoing surgical resection; (3) studies conducted in patients undergoing liver transplantation or bariatric surgery; and (4) studies investigating NAFLD and mortality from a certain disease (e.g., colorectal cancer). Based on the above-mentioned eligibility criteria, two investigators (J.J.H. and H.Y.T.) first scrutinized titles and abstracts to exclude apparently irrelevant studies, and then carefully reviewed the full text to further exclude unrelated studies. Any discrepancies regarding study eligibility were settled by discussion.

### Data extraction

The following information was extracted from eligible studies: first author’s family name, publication date, study location, study population, the source of cohort, mean age, follow-up duration, the number of NAFLD cases, sample size, the number of deaths, the approach to diagnose hepatic steatosis, outcome assessment, the most fully adjusted risk estimates with corresponding 95% CIs, and adjustment variables. One investigator (H.Y.T.) extracted the required data through an electronic spreadsheet, and then another investigator (J.J.H.) checked the data for accuracy. When multiple reports were derived from the same population-based cohort, we extracted data from the report with the longest follow-up duration and the largest sample size. Any discrepancies regarding the results of data extraction were settled by discussion.

### Quality assessment

Two investigators (H.Y.T. and J.J.H.) used the Newcastle-Ottawa quality assessment scale to independently perform the quality assessment for included studies^19^. This scale consists of eight items, which are categorized into three aspects (i.e., selection, comparability, and outcome). An individual study could earn a maximum of nine stars after evaluating its three aspects. In the present study, high-quality studies were defined as those earning ≥7 stars. Any discrepancies regarding the results of quality assessment were handled by discussion.

### Statistical analysis

Hazard ratio (HR) was employed as a common measure to evaluate the association between NAFLD and mortality. Liver-related mortality was defined as mortality from any liver disease, including hepatocellular carcinoma (HCC) and cirrhosis. Relative risk^7^ and standardized mortality ratio^9^ were directly treated as an equivalent to HR^20^. A random-effects model was employed to combine risk estimate from each individual study. The Hedges Q statistic (a *P* < 0.10 suggesting statistically significance) and the *I*^2^ statistic (an *I*^2^ of <50%, 50.0–75.0%, and >75.0% suggesting low, moderate and substantial heterogeneity, respectively) were employed to qualitatively and quantitatively assess the statistical heterogeneity among studies, respectively. For studies whose authors reported risk estimates by sex^14,21^, by the grade of NAFLD^22^, or by the number of comorbidities^7^, we combined these stratum data using a fixed-effects model to approximate risk estimates for the main analysis. Similarly, for one study whose authors reported risk estimates separately for NAFLD patients with and without increased levels of liver enzymes^13^, we combined these stratum data to obtain risk estimates for NAFLD patients.

We performed predefined subgroup analyses to investigate whether the observed association of NAFLD with all-cause and CVD mortality was modified by age, sex, study location, the number of NAFLD cases, NAFLD severity, presence of cirrhosis or fibrosis, methods to diagnose NAFLD, quality score, the source of cohort, follow-up duration and adjustment for body mass index, diabetes, smoking, hypertension, or hyperlipidemia/hypercholesterolemia. A *P*_interaction_ for the difference between subgroups was calculated through meta-regression. To identify possible sources of heterogeneity and to evaluate the stability of pooled results, we performed sensitivity analyses with the following approaches: using various exclusion criteria, ignoring a single study in turn, and repeating meta-analysis through a fixed-effects model. We employed Begg rank correlation test^23^ and Egger linear regression test^24^ to assess publication bias when there were ≥10 studies. We conducted all data analyses through STATA software (version12.0, StataCorp, College Station, TX), and adopted a statistical significance level of *P* < 0.05 under two-sided test unless otherwise specified.

## Results

### Literature search

**Figure 1:**
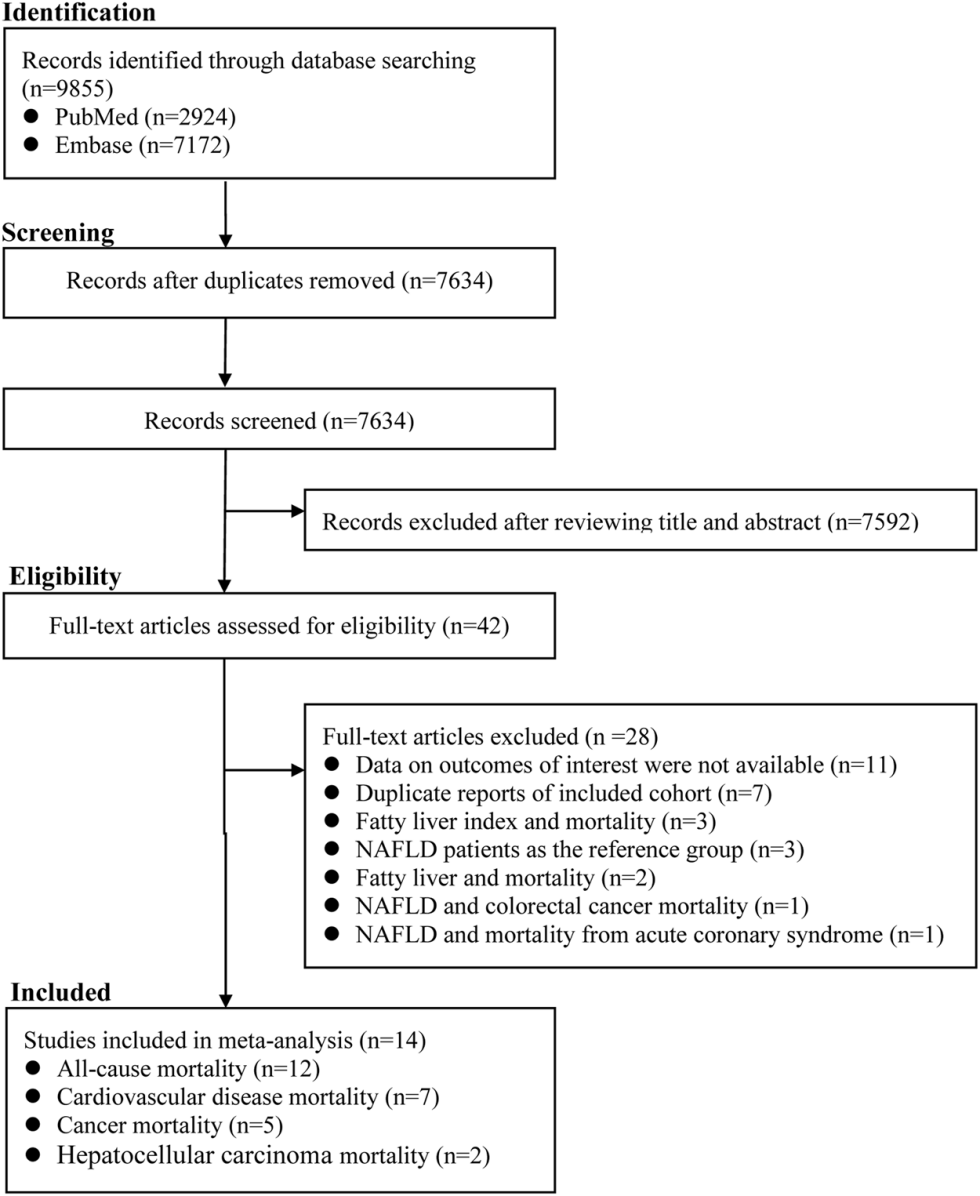
The flowchart of identifying relevant studies. NAFLD, nonalcoholic fatty liver disease.

The literature search identified 2924 and 7172 records from PubMed and Embase, respectively. After removing duplicates, we obtained a total of 7634 records. After carefully reviewing their titles and abstracts, we excluded a total of 7592 records. The remaining 42 records were assessed for eligibility through scrutinizing the full text. Of them, 28 were excluded for various reasons (Table S2 provided relevant information in detail). Of note, two reports deriving from the NHANES III cohort^12,13^ and two reports deriving from the Rochester Epidemiology Project^9,25^ were included, because their data were used for different analyses (the data reported by Lazo *et al*.^13^ and Adams *et al*.^9^ were used for analyses of all-cause mortality, and the data reported by Stepanova *et al*.^12^ and Adams *et al*.^25^ were used for analyses of CVD mortality). Thus, a total of 14 reports were included (Fig. 1).

### Study characteristics and quality assessment

Table S3 summarizes the main characteristics of included studies. Six studies were performed in Europe^6,8,10,21,22,26^, six in the USA^7,9,11–13,25^, one in South Korea^14^, and one in Australia^27^. The majority of included studies recruited their participants from the general population, while five studies recruited participants from the population with type 2 diabetes^6,25^, acute heart failure^26^, chronic kidney disease^10^, or myocardial infarction^22^. Of included studies, the number of NAFLD cases varied from 116^25^ to 82899^14^, yielding more than 95111 NAFLD cases; the sample size ranged from 264^26^ to 318224^14^, resulting in a total of 498501 participants. During follow-up period ranging from 1.9 years^26^ to 26.4 years^8^, a total of 24234 deaths occurred. As for quality assessment, half of included studies were scored ≥7 stars, with an average score of 6.5, suggesting that the quality of included studies was generally good (Supplementary Table 4).

### NAFLD and all-cause mortality

**Figure 2:**
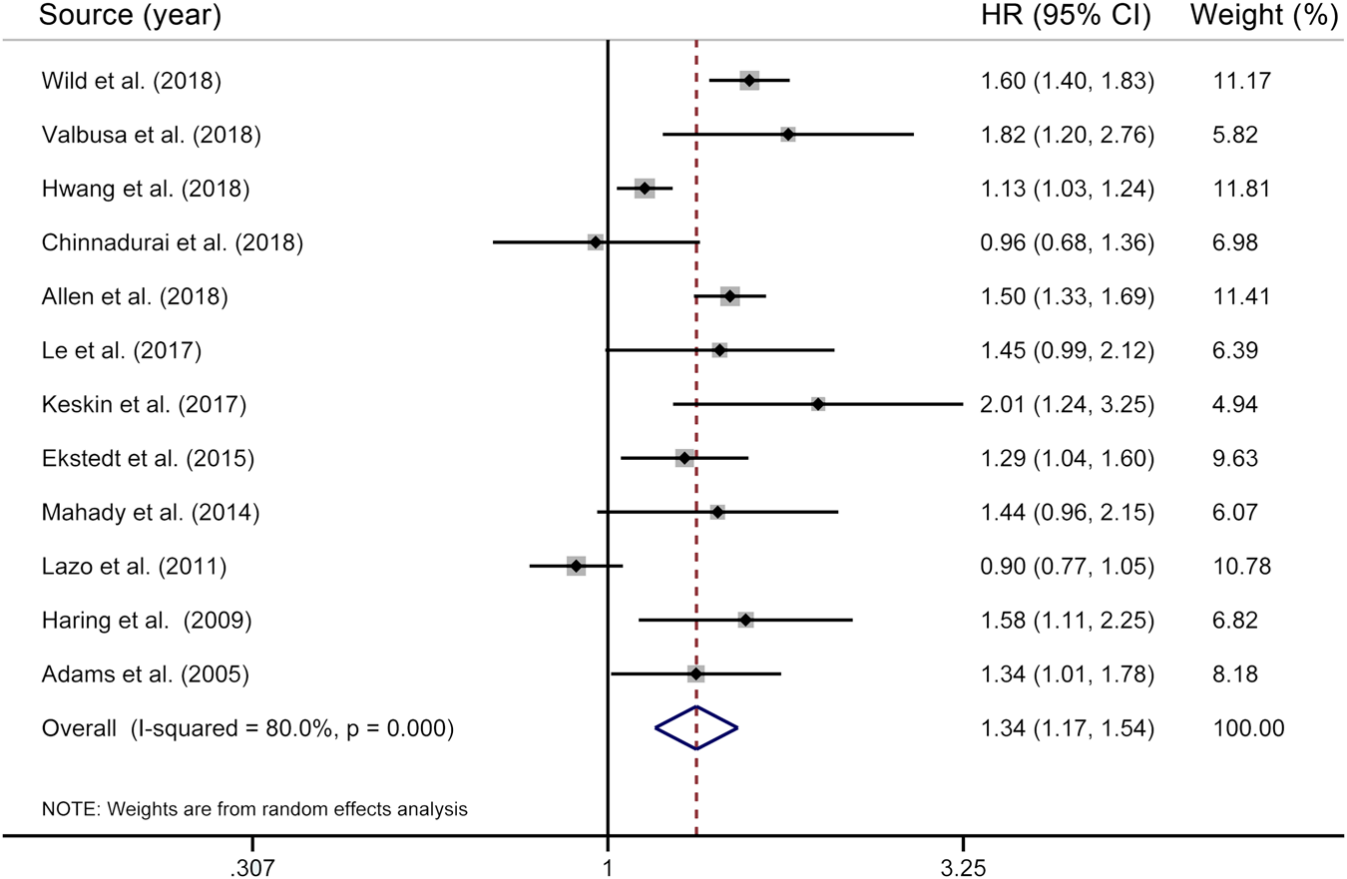
Results of meta-analysis on nonalcoholic fatty liver disease and all-cause mortality. The squares represent the risk estimate for each individual study, with the area reflecting the weight assigned to the study. The horizontal line across each square represents the 95% CI. The diamond represents the summary risk estimate, with width representing 95% CI. HR, hazard ratio; CI, confidence interval.

Our meta-analysis included a total of 12 individual studies^6–11,13,14,21,22,26,27^ for the association between NAFLD and all-cause mortality, involving 498259 participants and 24188 deaths. The pooled results showed that patients with NAFLD were at an increased risk of death from all causes compared with those without (HR = 1.34; 95% CI 1.17–1.54), with substantial heterogeneity (*I*^2^ = 80.0%, *P* < 0.01) (Fig. 2).

The results of subgroup analyses regarding NAFLD and all-cause mortality are shown in Table 1. We did not find evidence of significant effect modification by age, sex, study location, the number of NAFLD cases, NAFLD severity, presence of cirrhosis or fibrosis, methods to diagnose NAFLD, quality score, the source of cohort, follow-up duration, and adjustment for body mass index, diabetes, smoking, hypertension, or hyperlipidemia/hypercholesterolemia (all *P*_interaction_ > 0.05). Nevertheless, on the basis of limited studies, we found that NAFLD was significantly associated with an increased risk of all-cause mortality in women (four studies; HR = 1.49; 95% CI 1.15–1.93) but not in men (four studies; HR = 1.08; 95% CI 0.83–1.41). The results of sensitivity analyses are shown in Table 2. Ignoring a single study in turn did not materially alter the initial association between NAFLD and all-cause mortality (Fig. S1), with the pooled HRs ranging from 1.31 (95% CI 1.14–1.51)^6^ to 1.40 (95% CI 1.24–1.58)^13^. Similarly, repeating the analysis with a fixed-effects model did not materially alter the initial results. Moreover, the significant association of NAFLD with all-cause mortality remained after using various exclusion criteria. Of note, the exclusion of four studies with large sample size (>10000)^6,7,13,14^ yielded a similar risk estimate (HR = 1.40; 95% CI 1.22–1.61), but statistical heterogeneity significantly decreased (*I*^2^ changed from 80.0% to 25.0%). Both Begg’s test and Egger’s test did not find evidence of publication bias for the association of NAFLD with all-cause mortality (*P* > 0.05) (Fig. S2).

**Table 1:** Subgroup analyses of NAFLD and all-cause and cardiovascular disease mortality.

Subgroup		All-cause mortality								Cardiovascular disease mortality							
		n		HR (95% CI)		*I*^2^ (%)	*P*^a^		*P*^b^	n		HR (95% CI)		*I*^2^ (%)	*P*^a^		*P*^b^
All studies		12		1.34 (1.17–1.54)		80.0	<0.01		—	7		1.13 (0.92–1.38)		57.5	0.03		—
**Age (years)**																	
≥50		6		1.50 (1.31–1.73)		47.0	0.09		0.10	3		1.28 (0.87–1.88)		24.3	0.27		0.52
<50		6		1.20 (1.03–1.40)		69.2	0.01			4		1.08 (0.84–1.39)		71.4	0.02		
**Sex**																	
Male and Female		8		1.43 (1.29–1.59)		36.1	0.14		0.09	5		1.22 (0.93–1.61)		54.1	0.07		0.48
Male		4		1.08 (0.83–1.41)		75.3	0.01			2		0.93 (0.67–1.29)		60.6	0.11		
Female		4		1.49 (1.15–1.93)		64.6	0.04			2		1.25 (0.76–2.06)		56.1	0.13		
**Study location**																	
USA		4		1.26 (0.93–1.70)		88.9	<0.01		0.35	2		0.92 (0.71–1.19)		0	0.97		0.54
Europe		6		1.47 (1.23–1.75)		56.6	0.04			3		1.13 (0.79–1.61)		76.9	0.01		
**NAFLD cases**																	
≥1000		6		1.31 (1.08–1.59)		89.1	<0.01		0.68	4		1.01 (0.85–1.20)		41.6	0.16		0.07
<1000		6		1.38 (1.15–1.65)		41.8	0.13			3		1.56 (1.17–2.08)		0	0.42		
**NAFLD severity**																	
Simple hepatic steatosis		3		0.96 (0.83–1.12)		0.0	0.53		0.46	3		0.92 (0.78–1.10)		0.0	0.61		0.73
Nonalcoholic steatohepatitis		3		1.37 (0.77–2.43)		79.7	0.01			3		1.56 (1.17–2.08)		44.1	0.17		
**Presence of cirrhosis or fibrosis**																	
Yes		2		3.22 (2.40–4.33)		0.0	0.88		0.07	1		4.36 (2.29–8.29)		—	—		—
No		3		0.99 (0.67–1.46)		83.2	<0.01			1		1.28 (0.82–1.99)		—	—		
**Methods to diagnose NAFLD**																	
Ultrasonography		8		1.28 (1.07–1.54)		73.2	<0.01		0.61	4		1.05 (0.81–1.37)		64.3	0.04		0.48
Abdominal imaging or liver biopsy		3		1.52 (1.40–1.66)		0.0	0.50			2		1.13 (0.84–1.51)		0.0	0.66		
Liver biopsy		1		1.29 (1.04–1.60)		—	—			1		1.55 (1.11–2.16)		—	—		
**Quality score**																	
≥7		5		1.20 (0.99–1.45)		72.3	0.01		0.17	5		1.04 (0.82–1.32)		52.6	0.08		0.30
<7		7		1.45 (1.28–1.64)		49.7	0.06			2		1.33 (0.99–1.78)		40.6	0.20		
**Source of cohort**																	
Population-based		7		1.37 (1.12–1.66)		83.5	<0.01		0.79	5		1.01 (0.81–1.27)		43.1	0.13		0.19
Hospital-based		5		1.30 (1.06–1.59)		65.9	0.02			2		1.33 (1.04–1.70)		32.4	0.22		
**Follow-up duration (years)**																	
≥7		6		1.30 (1.05–1.61)		82.4	<0.01		0.61	5		1.13 (0.82–1.56)		69.1	0.01		0.84
<7		6		1.40 (1.13–1.75)		81.3	<0.01			2		1.18 (0.97–1.43)		0	0.83		
**Adjustment for body mass index**																	
Yes		6		1.25 (1.00–1.56)		87.3	<0.01		0.63	4		1.08 (0.92–1.26)		0	0.50		0.56
No		6		1.46 (1.33–1.60)		0	0.681			3		1.31 (0.77–2.24)		82.9	<0.01		
**Adjustment for diabetes**																	
Yes		5		1.31 (0.94–1.82)		80.4	<0.01		0.58	2		1.06 (0.81–1.37)		51.6	0.15		0.63
No		6		1.34 (1.16–1.54)		67.6	0.01			3		1.31 (0.77–2.24)		82.9	<0.01		
**Adjustment for smoking**																	
Yes		5		1.18 (0.94–1.48)		88.5	<0.01		0.59	3		1.08 (0.92–1.28)		11.6	0.32		0.66
No		7		1.47 (1.35–1.61)		0	0.574			4		1.24 (0.78–1.97)		74.7	0.01		
**Adjustment for hypertension**																	
Yes		6		1.28 (1.02–1.62)		88.2	<0.01		0.66	2		1.18 (0.97–1.43)		0	0.83		0.56
No		6		1.44 (1.32–1.58)		0	0.851			5		1.13 (0.82–1.56)		69.1	0.01		
**Adjustment for hyperlipidemia or hypercholesterolemia**																	
Yes		5		1.18 (0.94–1.48)		88.5	<0.01		0.59	2		1.18 (0.97–1.43)		0	0.83		0.56
No		7		1.47 (1.35–1.61)		0	0.574			5		1.13 (0.82–1.56)		69.1	0.01		

Abbreviations: CI, confidence interval; HR, hazard ratio.

^a^*P* for heterogeneity.

^b^*P* for interaction between subgroups with meta-regression.

**Table 2:** Sensitivity analyses of NAFLD and all-cause and cardiovascular mortality.

Categories	All-cause mortality			Cardiovascular mortality		
	n	HR (95% CI)	*I*^2^ (%)	n	HR (95% CI)	*I*^2^ (%)
Using a random-effects model to pool risk estimates of included studies	12	1.34 (1.17–1.54)	80.0	7	1.13 (0.92–1.38)	57.5
Using a fixed-effects model to pool risk estimates of included studies	12	1.28 (1.21–1.35)	80.0	7	1.09 (0.96–1.23)	57.5
Excluding studies using the general population as the reference group	9	1.34 (1.11–1.61)	82.7	6	1.05 (0.87–1.27)	44.7
Excluding studies using liver biopsy to diagnose NAFLD	8	1.28 (1.07–1.54)	73.2	4	1.05 (0.81–1.37)	64.3
Excluding studies conducted in the population with comorbidity	8	1.27 (1.10–1.48)	78.8	5	1.15 (0.89–1.49)	71.1
Excluding studies with sample size more than 10000	8	1.40 (1.22–1.61)	25.0	4	1.24 (0.78–1.97)	74.7
Excluding studies with sample size less than 1000	8	1.32 (1.12–1.55)	84.8	6	1.14 (0.92–1.41)	64.3
Excluding studies without full adjustment for confounders^a^	7	1.42 (1.17–1.73)	78.6	4	1.01 (0.85–1.20)	41.6

^a^Full adjustment refers to adjustment for age, sex and ≥3 important confounders (cardiovascular diseases, diabetes, smoking, alcohol, body mass index, physical activity and socioeconomic status as well as factors associated with these confounders.

### NAFLD and CVD mortality

**Figure 3:**
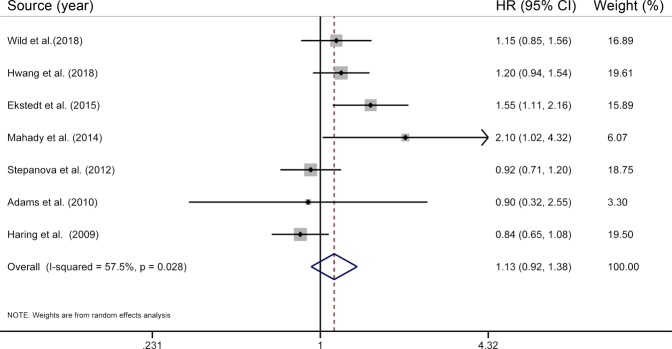
Results of meta-analysis on nonalcoholic fatty liver disease and cardiovascular disease mortality. The squares represent the risk estimate for each individual study, with the area reflecting the weight assigned to the study. The horizontal line across each square represents the 95% CI. The diamond represents the summary risk estimate, with width representing 95% CI. HR, hazard ratio; CI, confidence interval.

The present meta-analysis included a total of seven individual studies^6,8,12,14,21,25,27^ for the association of NAFLD with CVD mortality, involving 471849 subjects and more than 5541 deaths. Our study found that NAFLD was not significantly associated with the risk of death from CVD (HR = 1.13; 95% CI 0.92–1.38), with moderate heterogeneity (*I*^2^ = 57.5%, *P* = 0.03) (Fig. 3), and this finding could not be modified by predefined stratified factors (all *P*_interaction_ > 0.05) (Table 1). Pooling risk estimates with a fixed-effects model (HR = 1.09; 95% CI 0.96–1.23) and omitting each study in turn [the pooled HRs ranged from 1.05 (95% CI 0.87–1.23)^8^ to 1.20 (95% CI 0.98–1.46)^21^] did not alter the initial finding that there was no significant association between NAFLD and CVD mortality. In addition, the nonsignificant results on NAFLD and CVD mortality remained after using various exclusion criteria (Table 2).

### NAFLD and cancer mortality

**Figure 4:**
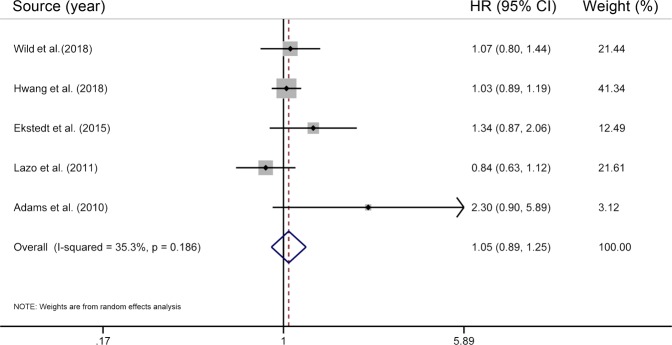
Results of meta-analysis on nonalcoholic fatty liver disease and overall cancer mortality. The squares represent the risk estimate for each individual study, with the area reflecting the weight assigned to the study. The horizontal line across each square represents the 95% CI. The diamond represents the summary risk estimate, with width representing 95% CI. HR, hazard ratio; CI, confidence interval.

The present study included five individual studies^6,8,13,14,25^ for the association between NAFLD and cancer mortality, involving a total of 465112 participants and more than 6924 deaths. The pooled results on NAFLD and cancer mortality are shown in Fig. 4. NAFLD was not significantly associated with cancer mortality (HR = 1.05; 95% CI 0.89–1.25), with low heterogeneity (*I*^2^ = 35.3%, *P* = 0.19). Both using a fixed-effects model (HR = 1.04; 95% CI 0.92–1.16) and ignoring a single study in turn did not materially alter the initial results on NAFLD and cancer mortality.

In addition, we investigated the association between NAFLD and mortality from HCC and non-HCC. Among included studies, two^6,8^ examined the association of NAFLD with HCC mortality, one^6^ examined the association of NAFLD with non-HCC mortality (HR = 0.76; 95% CI 0.55–1.04), and one^8^ examined the association of NAFLD with mortality from gastrointestinal malignancy (HR = 0.60; 95% CI 0.22–1.64) and nongastrointestinal malignancy (HR = 1.18; 95% CI 0.70–1.98). Based on two studies^6,8^ on NAFLD and HCC mortality, our meta-analysis found that patients with NAFLD had a 6.27-fold higher risk of death from HCC compared with those without (HR = 6.27; 95% CI 3.43–11.45; *I*^2^ = 0.0%, *P* = 0.93) (Fig. S3).

### NAFLD and liver-related mortality

**Figure 5:**
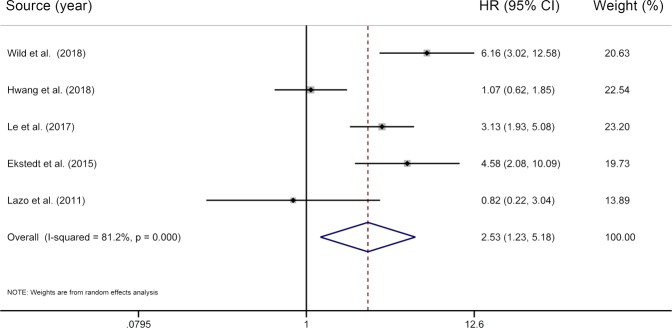
Results of meta-analysis on nonalcoholic fatty liver disease and liver-related mortality. The squares represent the risk estimate for each individual study, with the area reflecting the weight assigned to the study. The horizontal line across each square represents the 95% CI. The diamond represents the summary risk estimate, with width representing 95% CI. HR, hazard ratio; CI, confidence interval.

Our study included five individual studies^6,8,11,13,14^ for the association between NAFLD and liver-related mortality, involving more than 255 liver-related deaths among 470775 participants. Our meta-analysis found that NAFLD was associated with an increased risk of death from liver disease (HR = 2.53; 95% CI 1.23–5.18), with substantial heterogeneity (*I*^2^ = 81.2%, *P* < 0.01) (Fig. 5).

## Discussion

In recent years, there is a rapidly growing interest in determining whether NAFLD and its severity are associated with an increased risk of mortality. A recent meta-analysis found that NAFLD patients with fibrosis had a higher risk of all-cause mortality than those without, and such risk increased with increases in fibrosis stage^28^. However, whether patients with NAFLD are at an increased risk of mortality compared with those without is still under debate. Based on a total of 14 individual studies, involving 498501 subjects and 24234 deaths, our meta-analysis provided solid evidence for the association between NAFLD and mortality. Our results showed that NAFLD was associated with an increased risk of all-cause mortality but not CVD and cancer mortality. The observed association between NAFLD and all-cause and CVD mortality could not be modified by all prespecified stratified factors, and persisted in sensitivity analyses. Our findings on NAFLD and CVD mortality are consistent with those from a previous meta-analysis^5^.

However, our findings on NAFLD and all-cause mortality are inconsistent with those from two recent meta-analyses^1,15^. One meta-analysis in 2016 involving only four studies did not detect a significant association between NAFLD and overall mortality^1^. Of note, four studies included in this meta-analysis were all derived from the NHANES III cohort^13,16–18^. Similarly, another 2016 meta-analysis^15^ showing a null association of NAFLD with all-cause mortality included only five studies^8,13,16,17,25^, of which three were derived from the NHANES III cohort^13,16,17^. The inclusion of duplicate data from the same population-based cohort possibly produces biased estimates and exaggerated accuracy, and such methodological weakness is thought to be a critical threat to the validity of meta-analysis^29,30^. Therefore, in our meta-analysis, we paid much attention to the detection and exclusion of duplicate data. For example, a total of seven reports were found to originate from the NHANES III cohort (Table S2); however, only two of them were finally included in our meta-analysis after checking their end points^12,13^.

It is noteworthy that two key questions concerning the association of NAFLD with mortality remain unsolved in the present study. NAFLD is a heterogeneous disease that is comprised of a wide spectrum of histological conditions ranging from simple hepatic steatosis to NASH. Targher *et al*.^5^ found that the severity of NAFLD predicted both fatal CVD events alone and non-fatal and fatal CVD events combined. Thus, one question is whether the adverse effect of NAFLD on mortality is restricted to patients with NASH or can extend into those with simple hepatic steatosis. Solving the above-mentioned question is challenging, considering that liver biopsy is required for establishing a definite diagnosis of NASH. Indeed, there are few epidemiological studies that have addressed this question. A follow-up study of 229 patients with biopsy-proven NAFLD adopted NAFLD activity score, an index proposed by NASH Clinical Research Network^31^, to diagnose NASH (defined as NAFLD activity score >4); the results showed that there was no significant difference in overall and CVD mortality between patients with and without NASH^8^. Interestingly, a prospective cohort study, in which the grade of hepatic steatosis was classified into normal, mild, moderate and severe based on liver echogenicity, found that only moderate to severe steatosis was a predictor of increased all-cause mortality^32^. Based on few studies, our subgroup analysis by NAFLD severity seemed to suggest that only NASH was associated with an increased risk of death from all causes and CVD; however, this finding should be interpreted with much caution due to limited studies included. More studies are definitely needed to clarify this critical issue in the future. Another question is whether the direction and magnitude of the association between NAFLD and mortality can be modified by sex. In the present study, subgroup analysis by sex showed that NAFLD was significantly associated with an increased risk of all-cause mortality in women but not in men, although the risk difference between subgroups was not significant (*P*_interaction_ > 0.05). However, it should be reminded that these results were based on limited studies. Consequently, these results should be interpreted with caution, and need to be confirmed by large prospective cohort studies. If confirmed, identifying the mechanisms of action underlying the aforementioned risk difference between men and women appears to be interesting and challenging.

It is well established that CVD prevalence is significantly associated with NAFLD, and NAFLD patients have a higher risk of developing CVD than the general population^5^. CVD is the most frequent cause of death among NAFLD patients^33^. Interestingly, our meta-analysis suggested that NAFLD could not significantly increase the risk of death from CVD. The potential mechanism behind this phenomenon is unclear. A possible explanation may be that NAFLD patients are in a surveillance program, and benefit from an early and valid risk assessment for CVD, thereby decreasing CVD mortality to the level of the general population. Despite a null association between NAFLD and CVD mortality, our meta-analysis revealed a significant association between NAFLD and all-cause mortality. Considering the significant contribution of NAFLD to the burden of liver-related mortality^34^ as well as well-known hepatic complications among NAFLD patients, we argue that the increased all-cause mortality in NAFLD patients can be attributable to the increased liver-related mortality. Indeed, our meta-analysis found that patients with NAFLD had a higher risk of death from liver disease compared with those without. Nevertheless, our argument needs to be verified by future studies.

In the present study, we observed substantial heterogeneity across studies for the combined HR of NAFLD and all-cause mortality. As indicated by our sensitivity analyses, four studies with sample size more than 10000 are possible sources of the observed heterogeneity^6,7,13,14^. Notably, two of them recruited more than 100000 participants^6,14^. Thus, these large-scale studies would observe more deaths and yield more accurate risk estimates. Consequently, they were given more weight in the meta-analysis (all >10%). Moreover, larger studies are inclined to report smaller risk estimates and are conducted with more methodological rigor compared with smaller studies^35^. Indeed, if pooling risk estimates from the aforementioned four studies, we would obtain a smaller risk estimate (HR, 1.25; 95% CI, 0.99–1.58). In our subgroup analysis by age, we noticed that the statistical heterogeneity within subgroup obviously decreased, indicating that the age of subjects might also contribute to the observed heterogeneity. It has been shown that older adults have a significantly higher prevalence of NAFLD than young adults^2^. Therefore, the contribution of age to the observed heterogeneity is likely due to an imbalanced distribution of NAFLD prevalence among different age groups.

Our study has several limitations. First, we cannot assess the potential effect of dynamic changes in hepatic steatosis over time on the association between NAFLD and mortality, because all eligible studies did not provide data regarding repeat measurements of hepatic steatosis among their participants. As a result, our results might be subject to non-differential misclassification bias, which possibly attenuates the associations of interest. In addition, our results are based on study-level data; thus, we cannot perform an analysis of competing risk for cause-specific mortality to determine its potential impact on the corresponding results. Second, our study included five studies^6,10,22,25,26^ conducted in the population with preexisting conditions, which limits the generalizability of our results to some extent. Nonetheless, our sensitivity analysis showed that the initial results on the association of NAFLD with mortality remained after excluding the above-mentioned five studies. Third, although we extracted the fully adjusted risk estimates, we cannot rule out the potential impact of residual confounding on the pooled results, considering that our results were derived from observational studies where residual confounding always exists. Nonetheless, our subgroup analyses suggested that the observed association between NAFLD and overall and CVD mortality could not be modified by adjustment for body mass index, diabetes, smoking, hypertension, or hyperlipidemia/hypercholesterolemia, supporting that NAFLD by itself is a determinant for mortality. Finally, substantial heterogeneity was detected for the association between NAFLD and all-cause mortality. Nonetheless, we have identified sources of the observed heterogeneity through subgroup and sensitivity analyses. Moreover, clinical and methodological heterogeneity presents for all meta-analyses, especially meta-analysis of observational studies.

In conclusion, NAFLD is associated with an increased risk of all-cause mortality, while there are no significant associations of NAFLD with CVD and cancer mortality. These findings have important implications for decision making in public health and clinical practice, considering that NAFLD is highly prevalent across many countries and can lead to hepatic or extrahepatic complications. Moreover, our findings highlight that developing effective treatments for NAFLD is essential and should be regarded as a public health priority. Future studies should determine whether the observed association between NAFLD and mortality is limited to patients with NASH or can extend into those with simple hepatic steatosis.

## Supplementary information

Supplementary Information

## Data Availability

All data generated and analyzed during this study are included in this published article.
