# Supplementary material for: Nonalcoholic fatty liver disease and mortality from all causes, cardiovascular disease, and cancer: a meta-analysis
Source: Sci Rep. 2019 Jul 31;9:11124. doi: 10.1038/s41598-019-47687-3 (PMC6668400; doi:10.1038/s41598-019-47687-3)
Supplement: Supplementary file 1 — Supplementary Information [file 41598_2019_47687_MOESM1_ESM.pdf]

Nonalcoholic fatty liver disease and mortality from all causes, cardiovascular disease, and cancer: a meta-analysis

Yan Liu, Guo-Chao Zhong, Hao-Yang Tan, Fa-Bao Hao, Jie-Jun Hu

Supplementary Information

Table S1. Search Strategy for PubMed and Embase databases

Table S2. Excluded studies after reading the full text and corresponding reasons for exclusion

Table S3. Characteristics of included studies on NAFLD and mortality.

Table S4. Results of quality assessment of included studies

Figure S1. Sensitivity analysis on NAFLD and all-cause mortality: the exclusion of a single study in turn.

Figure S2. Results of Begg's test and Egger's test on the association of NAFLD with all-cause mortality.

Figure S3. Results of meta-analysis on nonalcoholic fatty liver disease and hepatocellular carcinoma mortality.

Table S1. Search Strategy for PubMed and Embase databases

Table S1. Search strategy for PubMed database (from its inception to October 17, 2018)

No.	Search strategy	Items found
#1	(((Non-alcoholic Fatty Liver Disease[MeSH Terms]) OR "fatty liver"[Title/Abstract]) OR steatohepatitis[Title/Abstract]) OR steatosis[Title/Abstract]	37313
#2	(((((death[MeSH Terms]) OR mortality[MeSH Terms]) OR death*[Title/Abstract]) OR mortalit*[Title/Abstract]) OR surviv*[Title/Abstract]) OR fatalit*[Title/Abstract]	2212493
#3	(review[Publication Type]) OR Published Erratum[Publication Type]) OR Editorial[Publication Type]	2941870
#4	#1 AND #2	4270
#5	#4 NOT #3	2924

Table S1-2. Search strategy for Embase database (from its inception to October 18, 2018)

No.	Search strategy	Items found
#1	'nonalcoholic fatty liver'/exp OR 'fatty liver':ab,ti OR 'steatohepatitis':ab,ti OR 'steatosis':ab,ti	63002
#2	'death'/exp OR 'mortality'/exp OR 'death*':ab,ti OR 'mortalit*':ab,ti OR 'surviv*':ab,ti OR 'fatalit*':ab,ti	3269906
#3	'review':it OR 'erratum':it OR 'editorial':it	3149973
#4	#1 AND #2	8921
#5	#4 NOT #3.	7172

Table S2. Excluded studies after reading the full text and corresponding reasons for exclusion.

Author	Year	Study description	Reasons for exclusion
Unalp-Arida et al. [1]	2018	This prospective study utilized NHANES III data, and investigated the association between liver fat scores and mortality. The authors explored the association between NAFLD liver fat score and mortality, but the reference group was patients with low NAFLD liver fat score rather than those without NAFLD.	NAFLD patients as the reference group.
Pais et al. [2]	2018	A prospective study on the association of hepatic steatosis with the risk of atherosclerosis.	Data on outcomes of interest were not available.
Chen et al. [3]	2018	This study recruited a total of 764 colorectal cancer patients undergoing primary surgical resection, and investigated the association between NAFLD and colorectal cancer mortality.	NAFLD and colorectal cancer mortality.
Yu et al. [4]	2017	A large cohort study investigated the effect of the number of metabolic	Data on outcomes of interest were

		risk factors on the risk of liver-related death. No risk estimates of NAFLD and mortality were found.	not available.
Brown et al. [5]	2017	This prospective study utilized NHANES III data, and investigated the association between NAFLD and mortality among cancer survivors.	Duplicate report of included cohort.
Zeb et al. [6]	2016	This prospective study investigated the association between NAFLD and incident cardiac event. The authors found that NAFLD was significantly associated with a composite endpoint inclusive of all-cause death and non-fatal CVD events.	Data on outcomes of interest were not available.
Unalp-Arida et al. [7]	2016	This prospective study utilized NHANES III data, and investigated the association between hepatic steatosis and mortality in the U.S. population.	Fatty liver and mortality.
Perera et al. [8]	2016	This descriptive study included a total of 120 patients with acute coronary syndrome, and investigated the association between NAFLD	NAFLD and mortality from acute coronary syndrome.

		and mortality from acute coronary syndrome.	
Seko et al. [9]	2015	This retrospective cohort study investigated the predictors of overall mortality in patients with NAFLD.	Data on outcomes of interest were not available.
Onat et al. [10]	2015	This prospective study employed fatty liver index to define fatty liver, and investigated the association between fatty liver and mortality.	Fatty liver index and mortality.
Zoppini et al. [11]	2014	This study investigated the association between chronic liver disease and mortality. However, it only provided risk estimates associated with non-virus, non-alcohol-related chronic liver disease rather than NAFLD.	Data on outcomes of interest were not available.
Pisto et al. [12]	2014	This population-based cohort study investigated the association between fatty liver and mortality.	Fatty liver and mortality.
Haflidadottir et al. [13]	2014	This retrospective study showed that patients with alcoholic fatty liver had higher liver-related mortality than those with NAFLD.	Data on outcomes of interest were not available.
Cheung et al. [14]	2014	This prospective study utilized NHANES III data, and investigated the	NAFLD patients as the reference

		association between NAFLD liver fat score and mortality. Moreover, the reference group was those with high NAFLD liver fat score.	group.
Otgonsuren et al. [15]	2013	This study utilized NHANES III data, and only provided risk estimates of NAFLD and liver-specific mortality.	Duplicate report of included cohort.
Lerchbaum et al. [16]	2013	This prospective cohort study included a total of 3270 patients referred to coronary angiography, and investigated the association between fatty liver index and mortality.	Fatty liver index and mortality.
Kim et al. [17]	2013	This prospective study utilized NHANES III data, and investigated the association between NAFLD and mortality. Its median follow-up duration was same as the study by Lazo et al. (2010) (14.5y), but its sample size (11154) was less than the study by Lazo et al. (2010) (11371).	Duplicate report of included cohort.
Zhou et al. [18]	2012	This prospective cohort study investigated the natural course of NAFLD	Data on outcomes of interest were

		in a Chinese population, and showed that patients with NAFLD had higher mortality rates than those without. However, this study did not provide specific risk estimates on NAFLD and mortality.	not available.
Calori et al. [19]	2011	This population-based study investigated the association between fatty liver index and mortality in the general population.	Fatty live index and mortality.
Soderberg et al. [20]	2010	This study showed that subjects with NAFLD had an increased risk of death compared with the general population. However, patients in this study were included in a recent study by Ekstedt et al. (2015).	Duplicate report of included cohort.
Yun et al. [21]	2009	This prospective study investigated the association between alanine aminotransferase levels and mortality from cardiovascular disease or diabetes in a Korean population.	Data on outcomes of interest were not available.
Ong et al. [22]	2008	This prospective study utilized NHANES III data, and investigated the association between NAFLD and mortality. Of note, both sample size	Duplicate report of included cohort.

		(11285) and follow-up duration (8.7y) of this study were less than the study by Lazo et al. (2010).	
Dunn et al. [23]	2008	This prospective study utilized NHANES III data, and investigated the association between NAFLD and mortality. Of note, both sample size (7574) and follow-up duration (8.7y) of this study were less than the study by Lazo et al. (2010).	Duplicate report of included cohort.
Targher et al. [24]	2007	This prospective study investigated the association between NAFLD diagnosed by ultrasound and incident cardiovascular disease events. In this study, cardiovascular disease events were defined as myocardial infarction, ischemic stroke, coronary revascularization, or cardiovascular death.	Data on outcomes of interest were not available.
Ekstedt et al. [25]	2006	This study showed that patients with NAFLD did not have an increased mortality compared with the general population. However, patients in	Duplicate report of included cohort.

		this study were included in a recent study by Ekstedt et al. (2015).	
Targher et al. [26]	2005	This prospective nested case-control study investigated the association between NAFLD and cardiovascular disease events. However, it did not report risk estimates of mortality from cardiovascular disease.	Data on outcomes of interest were not available.
Jepsen et al. [27]	2003	This retrospective cohort study provided standardized mortality ratio for patients with non-alcoholic or unspecified fatty liver, but it did not provide risk estimates for those with NAFLD separately.	Data on outcomes of interest were not available.
Matteoni et al. [28]	1999	This study compared clinical characteristics and outcomes of patients with different types of NAFLD. The reference group was patients with NAFLD.	NAFLD patients as the reference group.

Abbreviations: NHANES III, the third National Health and Nutrition Examination Survey; NAFLD, nonalcoholic fatty liver disease; NASH, nonalcoholic steatohepatitis.

References

1. Unalp-Arida A, Ruhl CE. Liver fat scores predict liver disease mortality in the United States population. Alimentary pharmacology &

therapeutics 2018.

2. Pais R, Redheuil A, Cluzel P, Ratziu V, Giral P. Relationship between fatty liver, specific and multiple-site atherosclerosis and 10-year Framingham Score. Hepatology (Baltimore, Md) 2018.

3. Chen ZF, Dong XL, Huang QK, Hong WD, Wu WZ, Wu JS, et al. The combined effect of non-alcoholic fatty liver disease and metabolic syndrome on colorectal carcinoma mortality: a retrospective in Chinese females. World journal of surgical oncology 2018; 16 (1):163.

4. Yu MW, Lin CL, Liu CJ, Yang SH, Tseng YL, Wu CF. Influence of Metabolic Risk Factors on Risk of Hepatocellular Carcinoma and Liver-Related Death in Men With Chronic Hepatitis B: A Large Cohort Study. Gastroenterology 2017; 153 (4):1006-1017 e1005.

5. Brown JC, Harhay MO, Harhay MN. Nonalcoholic fatty liver disease and mortality among cancer survivors. Cancer epidemiology 2017; 48:104-109.

6. Zeb I, Li D, Budoff MJ, Katz R, Lloyd-Jones D, Agatston A, et al. Nonalcoholic Fatty Liver Disease and Incident Cardiac Events: The Multi-Ethnic Study of Atherosclerosis. Journal of the American College of Cardiology 2016; 67 (16):1965-1966.

7. Unalp-Arida A, Ruhl CE. Noninvasive fatty liver markers predict liver disease mortality in the U.S. population. Hepatology (Baltimore, Md) 2016; 63 (4):1170-1183.

8. Perera N, Indrakumar J, Abeysinghe WV, Fernando V, Samaraweera WM, Lawrence JS. Non alcoholic fatty liver disease increases the mortality from acute coronary syndrome: an observational study from Sri Lanka. BMC cardiovascular disorders 2016; 16:37.

9. Seko Y, Sumida Y, Tanaka S, Taketani H, Kanemasa K, Ishiba H, et al. Predictors of malignancies and overall mortality in Japanese patients with biopsy-proven non-alcoholic fatty liver disease. Hepatology research : the official journal of the Japan Society of Hepatology 2015; 45 (7):728-738.

10. Onat A, Can G, Kaya A, Akbas T, Ozpamuk-Karadeniz F, Simsek B, et al. Fatty liver disease: Disparate predictive ability for cardiometabolic risk and all-cause mortality. World journal of gastroenterology 2015; 21 (48):13555-13565.

11. Zoppini G, Fedeli U, Gennaro N, Saugo M, Targher G, Bonora E. Mortality from chronic liver diseases in diabetes. The American journal of gastroenterology 2014; 109 (7):1020-1025.

12. Pisto P, Santaniemi M, Bloigu R, Ukkola O, Kesaniemi YA. Fatty liver predicts the risk for cardiovascular events in middle-aged population: a population-based cohort study. BMJ open 2014; 4 (3):e004973.

13. Haflidadottir S, Jonasson JG, Norland H, Einarsdottir SO, Kleiner DE, Lund SH, et al. Long-term follow-up and liver-related death rate in patients with non-alcoholic and alcoholic related fatty liver disease. BMC gastroenterology 2014; 14:166.

14. Cheung CL, Lam KS, Wong IC, Cheung BM. Non-invasive score identifies ultrasonography-diagnosed non-alcoholic fatty liver disease and predicts mortality in the USA. BMC medicine 2014; 12:154.

15. Otgonsuren M, Stepanova M, Gerber L, Younossi ZM. Anthropometric and clinical factors associated with mortality in subjects with nonalcoholic fatty liver disease. Digestive diseases and sciences 2013; 58 (4):1132-1140.

16. Lerchbaum E, Pilz S, Grammer TB, Boehm BO, Stojakovic T, Obermayer-Pietsch B, et al. The fatty liver index is associated with increased mortality in subjects referred to coronary angiography. Nutrition, metabolism, and cardiovascular diseases : NMCD 2013; 23 (12):1231-1238.

17. Kim D, Kim WR, Kim HJ, Therneau TM. Association between noninvasive fibrosis markers and mortality among adults with nonalcoholic fatty liver disease in the United States. Hepatology (Baltimore, Md) 2013; 57 (4):1357-1365.

18. Zhou YJ, Li YY, Nie YQ, Huang CM, Cao CY. Natural course of nonalcoholic fatty liver disease in southern China: a prospective cohort study. Journal of digestive diseases 2012; 13 (3):153-160.

19. Calori G, Lattuada G, Ragogna F, Garancini MP, Crosignani P, Villa M, et al. Fatty liver index and mortality: the Cremona study in the 15th year of follow-up. Hepatology (Baltimore, Md) 2011; 54 (1):145-152.

20. Soderberg C, Stal P, Askling J, Glaumann H, Lindberg G, Marmur J, et al. Decreased survival of subjects with elevated liver function tests

during a 28-year follow-up. Hepatology (Baltimore, Md) 2010; 51 (2):595-602.

21. Yun KE, Shin CY, Yoon YS, Park HS. Elevated alanine aminotransferase levels predict mortality from cardiovascular disease and diabetes in Koreans. Atherosclerosis 2009; 205 (2):533-537.

22. Ong JP, Pitts A, Younossi ZM. Increased overall mortality and liver-related mortality in non-alcoholic fatty liver disease. Journal of hepatology 2008; 49 (4):608-612.

23. Dunn W, Xu R, Wingard DL, Rogers C, Angulo P, Younossi ZM, et al. Suspected nonalcoholic fatty liver disease and mortality risk in a population-based cohort study. The American journal of gastroenterology 2008; 103 (9):2263-2271.

24. Targher G, Bertolini L, Rodella S, Tessari R, Zenari L, Lippi G, et al. Nonalcoholic fatty liver disease is independently associated with an increased incidence of cardiovascular events in type 2 diabetic patients. Diabetes care 2007; 30 (8):2119-2121.

25. Ekstedt M, Franzen LE, Mathiesen UL, Thorelius L, Holmqvist M, Bodemar G, et al. Long-term follow-up of patients with NAFLD and elevated liver enzymes. Hepatology (Baltimore, Md) 2006; 44 (4):865-873.

26. Targher G, Bertolini L, Poli F, Rodella S, Scala L, Tessari R, et al. Nonalcoholic fatty liver disease and risk of future cardiovascular events among type 2 diabetic patients. Diabetes 2005; 54 (12):3541-3546.

27. Jepsen P, Vilstrup H, Mellemkjaer L, Thulstrup AM, Olsen JH, Baron JA, et al. Prognosis of patients with a diagnosis of fatty liver--a registry-based cohort study. Hepato-gastroenterology 2003; 50 (54):2101-2104.

28. Matteoni CA, Younossi ZM, Gramlich T, Boparai N, Liu YC, McCullough AJ. Nonalcoholic fatty liver disease: a spectrum of clinical and pathological severity. Gastroenterology 1999; 116 (6):1413-1419.

Table S3. Characteristics of included studies on NAFLD and mortality

Source; study location	Study population	Mean age (y)	Mean follow-up duration (y)	NAFLD cases/ sample size	Deaths	NAFLD diagnosis	Outcome assessment	Adjustment factors
Wild et al. (2018) [6]; UK	Population with type 2 diabetes	62.6	4.7	1452/ 132661	All-cause: 16424<br>CVD: 4469<br>cancer: 5673	Abdominal imaging or liver biopsy	National mortality records	Age, sex, antihypertensive treatment, cholesterol level, CVD history, glycated hemoglobin, hypertension, lipid-lowering treatment, smoking, and socioeconomic status
Valbusa et al. (2018) [26]; Italy	Population with acute heart failure	83.3	1.9	153/264	All-cause: 116	Ultrasonography	Contacting patients, family members, or patients' physician	Age, sex, body weight, chronic kidney disease, chronic obstructive pulmonary disease, coronary heart disease, diabetes, furosemide doses, γ- glutamyltransferase, history of heart failure, hospital ward, left ventricular ejection fraction, NT pro-brain natriuretic peptide, plasma albumin, systolic blood pressure, and use of

								antihypertensive drugs
Hwang et al. (2018) [14]; South Korea	General population	39.3	5.7 [1]	82899/ 318224	All-cause: 1613<br>CVD: 242<br>cancer: 748	Ultrasonography	Death certificate	Age, alcohol consumption, BMI, smoking, and physical activity (plus diabetes, hypertension, and hypercholesterolemia for CVD mortality only)
Chinnadurai et al. (2018) [10]; UK	Population with chronic kidney disease	66.0 [a]	5.4 [1]	183/852	All-cause: 271	Ultrasonography	Electronic patient records and National Statistic Office	Age, sex, BMI, cerebrovascular accidents, congestive cardiac failure, diabetes, diastolic blood pressure, estimated glomerular filtration rate, hyperlipidemia, hypertension, ischemic heart disease, malignancy, myocardial infarction, peripheral vascular disease, smoking, and systolic blood pressure
Allen et al. (2018) [7]; USA	General population	53.0 [a]	7.0 [1]	3869/19078	All-cause: 1551	Abdominal imaging or liver biopsy	Death certificate	(None, controls were matched to cases for age and sex)
Le et al. (2017) [11]; USA	General population	49.2	6.6	1936/6000	All-cause: 405	Ultrasonography	Death certificate	Age, sex, BMI, diabetes, education, metabolic syndrome, race, and

								smoking
Keskin et al. (2017) [22]; Turkey	Population with myocardial infarction	59.0	2.6	191/360	All-cause: 29	Ultrasonography	National Death Declaration system or follow-up interview	Age, sex, blood urea nitrogen, BMI, chronic kidney disease, C-reactive protein, creatine kinase, creatinine, diabetes, heart rate, hematocrit, hypertension, lymphocyte, medications, neutrophil, platelet count, SYNTAX score, systolic blood pressure, troponin I, waist circumference, and white blood cells
Ekstedt et al. (2015) [8]; Sweden	General population	48.8	26.4	229/2519	All-cause: 882<br>CVD: NA<br>cancer: NA	Liver biopsy	Death registry	(None, controls were matched to cases for age and sex)
Mahady et al. (2014) [27]; Australia	General population	70.0	11.0	NA/2335	All-cause: 701<br>CVD: 203	Ultrasonography	National Death Index	Age, sex, and alcohol consumption
Stepanova et al. (2012) [12]; USA	General population	(20.0 -74.0)	14.3	2492/11613	CVD: 591	Ultrasonography	Death certificate	Age, sex, diabetes, family history of myocardial infarction, obesity, race, and smoking
Lazo et al. (2011) [13];	General population	42.7	14.5 [1]	2515/11371	All-cause: 1836<br>CVD: 716	Ultrasonography	Death certificate	Sex, alcohol consumption, BMI, diabetes, education,

USA					cancer: 480			hypercholesterolemia, hypertension, physical activity, race, and smoking
Adams et al. (2010) [25]; USA	Population with type 2 diabetes	58.0	10.9	116/337	All-cause: 99<br>CVD: 36<br>cancer: 23	Abdominal imaging or liver biopsy	Death certificate	Age, sex, date of diabetes diagnosis, and obesity
Haring et al. (2009) [21]; Germany	General population	49.6	7.3 [a]	1249/4160	All-cause: 307<br>CVD: NA	Ultrasonography	Death certificate	Age, alcohol consumption, civil status, education, equalized income, functional comorbidity index, physical activity, and waist circumference
Adams et al. (2005) [9]; USA	General population	49.0	7.6	435/435	All-cause: 53	Abdominal imaging or liver biopsy	Death certificate	(None, controls were matched to cases for age and sex)

Abbreviations: BMI, body mass index; CVD, cardiovascular disease; NA, not available; NAFLD, nonalcoholic fatty liver disease.

[1] Median value.

Table S4 The results of quality assessment of included studies

Study	Selection				Comparability		Outcome			Total Score
	Representativeness of exposed cohort ☆	Selection of non-exposed cohort ☆	Exposure ascertainment ☆	No history of disease ☆	Comparable on confounders ☆☆		Outcome Assessment ☆	Adequate follow-up (≥10y) ☆	Loss to follow-up rate (≤20%) ☆	
Wild *et al*. (2018) [6]		☆	☆		☆	☆	☆		☆	6
Valbusa *et al*. (2018) [26]		☆	☆		☆	☆			☆	5
Hwang *et al*. (2018) [14]	☆	☆	☆	☆	☆	☆	☆		☆	8
Chinnadurai *et al*. (2018) [10]		☆	☆		☆	☆	☆			5
Allen *et al*. (2018) [7]	☆	☆	☆	☆			☆		☆	6
Le *et al*. (2017) [11]	☆	☆	☆	☆	☆	☆	☆		☆	8
Keskin *et al*. (2017) [22]		☆	☆		☆	☆			☆	5
Ekstedt *et al*. (2015) [8]		☆	☆	☆			☆	☆	☆	6
Mahady *et al*. (2014) [27]	☆	☆	☆	☆	☆		☆	☆		7
Stepanova *et al*. (2012) [12]	☆	☆	☆	☆	☆	☆	☆	☆		8
Lazo *et al*. (2011) [13]	☆	☆	☆	☆	☆		☆	☆		7
Adams *et al*. (2010) [25]		☆	☆	☆	☆		☆	☆	☆	7
Haring *et al*. (2009) [21]	☆	☆	☆	☆	☆	☆	☆		☆	8
Adams *et al*. (2005) [9]		☆	☆	☆			☆		☆	5

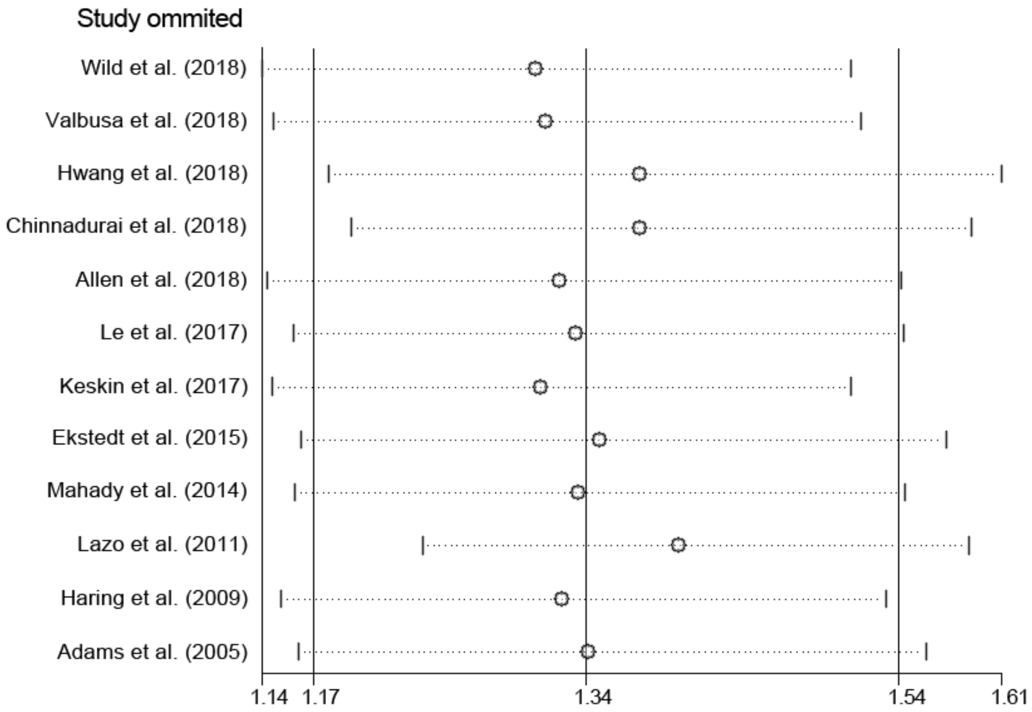

Figure S1. Sensitivity analysis on NAFLD and all-cause mortality: the exclusion of a single study in turn. The study cited on the left is the one left out in each turn. The circle represents the summary risk estimates after exclusion of a single study, and the corresponding dot line represents 95% confidence interval. The middle vertical solid line represents summary risk estimates of all included studies, and left and right vertical solid line represent lower limit and upper limit, respectively

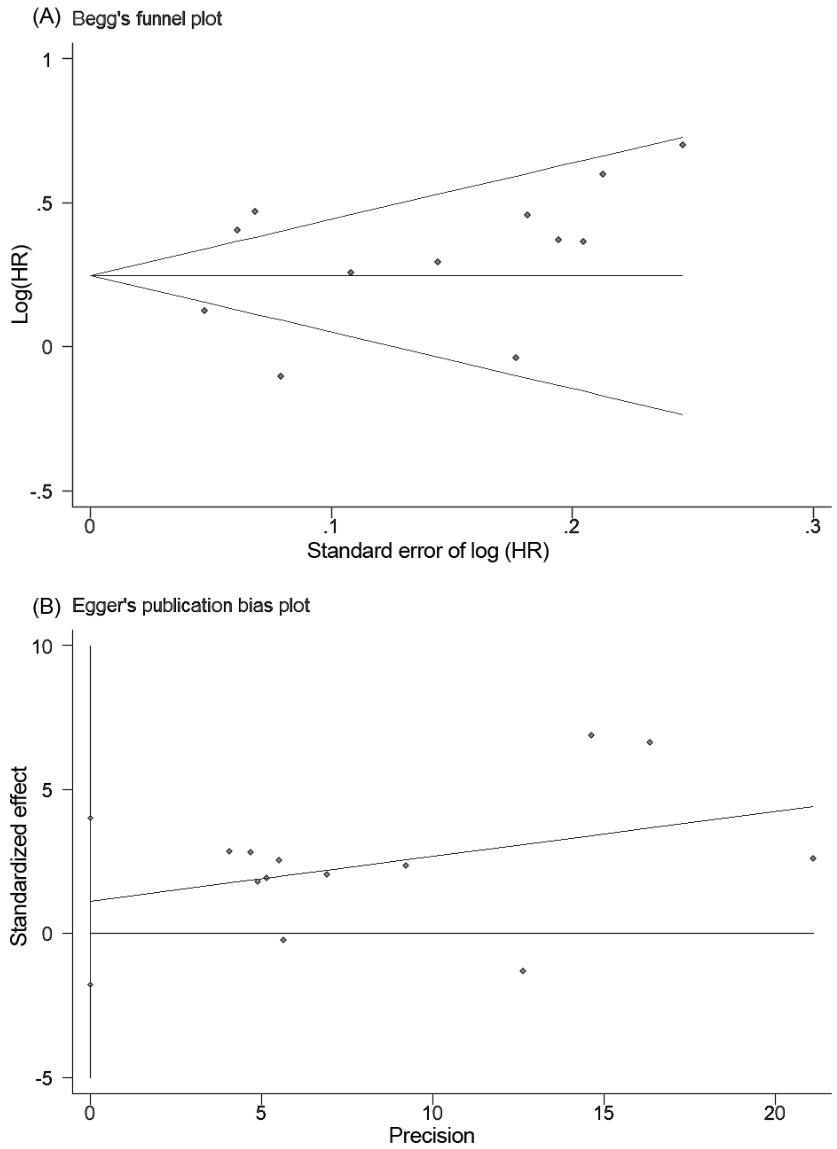

Figure S2. Results of Begg's test and Egger's test on the association of NAFLD with all-cause mortality

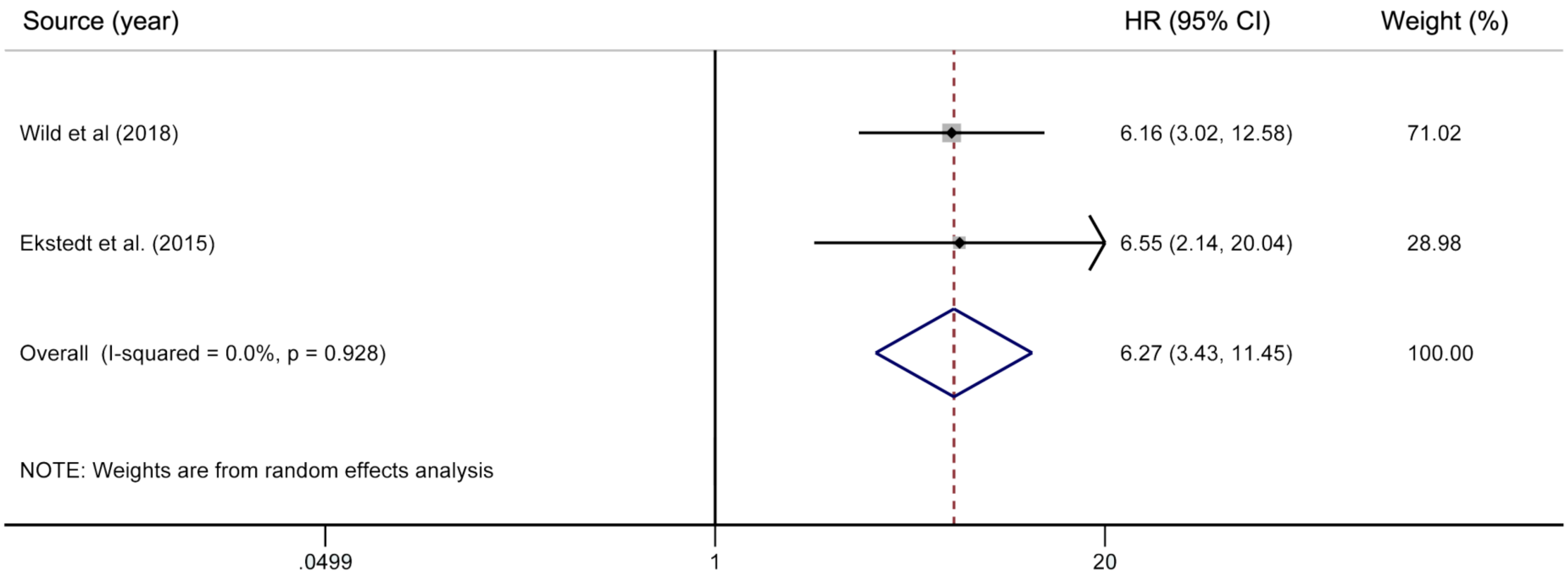

Figure S3. Results of meta-analysis on nonalcoholic fatty liver disease and hepatocellular carcinoma mortality. The squares represent the risk estimate for each individual study, with the area reflecting the weight assigned to the study. The horizontal line across each square represents the 95% CI. The diamond represents the summary risk estimate, with width representing 95% CI. HR, hazard ratio; CI, confidence interval.
